# Exendin-4 Ameliorates Motor Neuron Degeneration in Cellular and Animal Models of Amyotrophic Lateral Sclerosis

**DOI:** 10.1371/journal.pone.0032008

**Published:** 2012-02-23

**Authors:** Yazhou Li, Srinivasulu Chigurupati, Harold W. Holloway, Mohamed Mughal, David Tweedie, Daniel A. Bruestle, Mark P. Mattson, Yun Wang, Brandon K. Harvey, Balmiki Ray, Debomoy K. Lahiri, Nigel H. Greig

**Affiliations:** 1 Laboratory of Neurosciences, Intramural Research Program, National Institute on Aging, National Institutes of Health, Baltimore, Maryland, United States of America; 2 Molecular Neuropsychiatry Branch, Intramural Research Program, National Institute on Drug Abuse, National Institutes of Health, Baltimore, Maryland, United States of America; 3 Department of Psychiatry, Institute of Psychiatric Research, Indiana University School of Medicine, Indianapolis, Indiana, United States of America; University of Nebraska Medical center, United States of America

## Abstract

Amyotrophic lateral sclerosis (ALS) is a devastating neurodegenerative disease characterized by a progressive loss of lower motor neurons in the spinal cord. The incretin hormone, glucagon-like peptide-1 (GLP-1), facilitates insulin signaling, and the long acting GLP-1 receptor agonist exendin-4 (Ex-4) is currently used as an anti-diabetic drug. GLP-1 receptors are widely expressed in the brain and spinal cord, and our prior studies have shown that Ex-4 is neuroprotective in several neurodegenerative disease rodent models, including stroke, Parkinson's disease and Alzheimer's disease. Here we hypothesized that Ex-4 may provide neuroprotective activity in ALS, and hence characterized Ex-4 actions in both cell culture (NSC-19 neuroblastoma cells) and in vivo (SOD1 G93A mutant mice) models of ALS. Ex-4 proved to be neurotrophic in NSC-19 cells, elevating choline acetyltransferase (ChAT) activity, as well as neuroprotective, protecting cells from hydrogen peroxide-induced oxidative stress and staurosporine-induced apoptosis. Additionally, in both wild-type SOD1 and mutant SOD1 (G37R) stably transfected NSC-19 cell lines, Ex-4 protected against trophic factor withdrawal-induced toxicity. To assess in vivo translation, SOD1 mutant mice were administered vehicle or Ex-4 at 6-weeks of age onwards to end-stage disease via subcutaneous osmotic pump to provide steady-state infusion. ALS mice treated with Ex-4 showed improved glucose tolerance and normalization of behavior, as assessed by running wheel, compared to control ALS mice. Furthermore, Ex-4 treatment attenuated neuronal cell death in the lumbar spinal cord; immunohistochemical analysis demonstrated the rescue of neuronal markers, such as ChAT, associated with motor neurons. Together, our results suggest that GLP-1 receptor agonists warrant further evaluation to assess whether their neuroprotective potential is of therapeutic relevance in ALS.

## Introduction

Amyotrophic lateral sclerosis (ALS), also known as Lou Gehrig's disease and motor neuron disease, is an incurable neurodegenerative disorder of the voluntary motor system. Characterized by selective and progressive death of motor neurons within the brain and spinal cord, it leads to paralysis of voluntary muscles and, eventually, death within five years of clinical onset [1], [2]. Although most cases of ALS occur sporadically with unknown etiology, approximately 10% are inherited in an autosomal dominant manner [3]. Of these, 20% are caused by mutations within the gene encoding the superoxide dismutase 1 (SOD-1) protein, an enzyme involved in the detoxification of reactive oxygen species. Transgenic mice expressing the same SOD1 mutations as human, in particular the G93A point mutation, exhibit similar histopathological and clinical phenotypes as ALS patients [4], [5], and hence have been widely used to elucidate mechanisms inducing ALS pathology as well as to screen for potential therapeutics [4]. Presently, however, riluzole, an anti-excitotoxic agent that reduces the release of presynaptic glutamate, is the sole agent approved for ALS treatment [1], [2], [6]. Whereas riluzole provides some survival benefit, extending lifespan by 3–5 months, it does not significantly modify muscle strength or functional outcome. Hence new medications are needed to maintain the survival of motor neurons and slow the decline in independent function for patients with this incurable disease [1].

The endogenous incretin, glucagon-like peptide-1 (GLP-1), is a 30 amino acid hormone that stimulates glucose-dependent insulin secretion and inhibits both glucagon secretion and gastric emptying following food ingestion [7]. The long-acting GLP-1 receptor (GLP-1R) agonists exendin-4 (Ex-4) and liraglutide are approved therapeutics for the treatment of type 2 diabetes mellitus (T2DM) [8]. Administered and well tolerated subcutaneously (s.c.), they effectively lower blood glucose levels during hyperglycemia, but not euglycemia, and can hence be safely given to nondiabetic subjects [7], [8].

In addition to their presence on pancreatic β-cells, GLP-1 receptors (GLP-1R) are widely distributed and present on neurons within the brain and peripheral nervous system [7], [9]–[11]. GLP-1 and analogues have been reported to cross the blood-brain barrier [12], [13] and act as neurotrophic factors in the brain, inducing neurite outgrowth [14] as well as tyrosine hydroxylase expression [15], [16]. Similar to the action of Ex-4 on pancreatic β-cells, GLP-1 analogues have demonstrated anti-apoptotic actions in neuronal cultures; thereby providing protection against oxidative stress, hypoxia and trophic factor withdrawal [14], [15], [17]. Such actions appear to effectively translate to in vivo, where GLP-1 and analogues have reduced brain damage and improved functional outcome in a transient middle cerebral artery occlusion stroke model [15], [18], as well as in multiple animal models of Parkinson's disease (PD), induced by MPTP, 6-OHDA or LPS [15], [19]–[21]. Likewise, GLP-1 analogues have demonstrated favorable actions in rodent models of peripheral neuropathy [22] as well as Alzheimer's disease (AD) [17], [23]. In addition to neuroprotection, an elevation in neuronal progenitor cells was evident within the brain of GLP-1 agonist-treated animals [19], [20], [23] indicative of the induction of neuroregenerative processes.

The brain is a highly insulin sensitive organ and T2DM is known as a risk factor for many neurodegenerative diseases, including AD and PD in which the repositioning of Ex-4 is already being assessed as a new clinical treatment strategy. As glucose intolerance and insulin resistance have been linked to ALS [24], [25], and in light of the neurotrophic and protective actions of GLP-1R activation in diverse cellular and animal models of neurodegeneration, we hypothesize that GLP-1 and analogues may provide neuroprotective actions in ALS. To test this hypothesis, we investigated the therapeutic potential of Ex-4 in both cell culture and rodent models of ALS.

## Results

### GLP-1R is present in NSC19 cells and in mouse spinal cord

**Figure 1:**
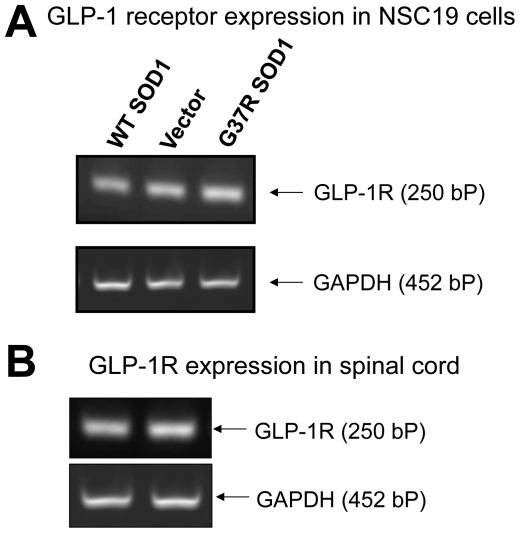
GLP-1R is expressed in NSC19 cells and mouse spinal cord. (**A**) A representative RT-PCR analysis showing expression of GLP-1R in stable transfected NSC19 cell lines (from left to right: NSC19 cells expressing wildtype SOD1, NSC19 cells expressing empty vector, NSC19 cells expressing G37R mutated SOD1). (**B**) A representative RT-PCR analysis showing expression of GLP-1R in wildtype C57Bl/6 mouse spinal cord (2 lanes represent 2 different mice). Glyceraldehyde 3-phosphate dehydrogenase (GAPDH) was utilized as an internal control. Using specific primers, amplified mouse GLP-1R fragment is 250 bp and GAPDH is 452 bp.

NSC19 is an immortal mouse neural cell line that exhibits specific features of spinal cord motor neurons [26], including the expression of the cholinergic marker choline acetyl transferase (ChAT), thereby providing a relevant cell culture model for the study of motor neuron biology and disorders such as ALS. In order to study mutant SOD1-mediated familial ALS, we established three stable NSC19 cell lines that expressed vector (as a control), wild-type (WT) SOD1 or mutated SOD1 (G37R), respectively. Prior to examining GLP-1R signaling in these cells, GLP-1R expression was first characterized. As illustrated in Figure 1A, GLP-1R mRNA is clearly present in all three NSC19 cell lines, as assessed by RT-PCR with specific mouse GLP-1R primers. In addition, to both aid translation to animal studies and validate the value NSC19 cells as an appropriate model of mouse motor neurons, total RNA was isolated from the spinal cord of wild-type C57Bl/6 mice and examined for the presence of GLP-1R mRNA by RT-PCR. Figure 1B demonstrates the presence of the GLP-1R in mouse spinal cord.

### GLP-1R stimulation is neurotrophic and neuroprotective against oxidative stress and apoptosis in NSC19 cells

**Figure 2:**
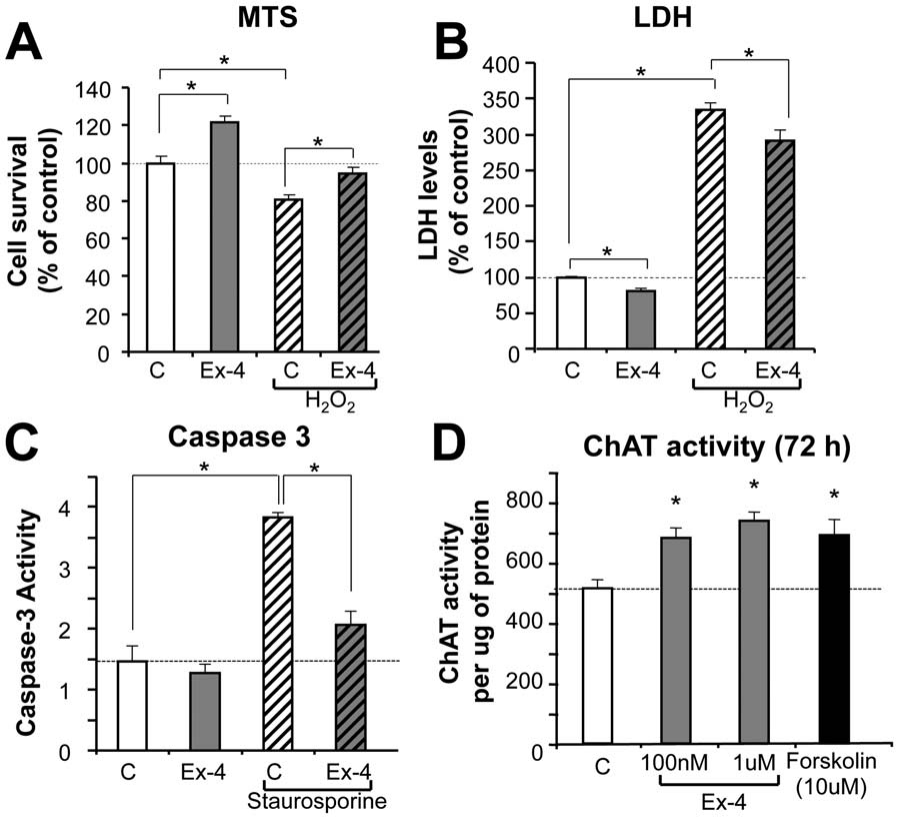
GLP-1R stimulation is neurotrophic and neuroprotective against oxidative stress and apoptosis in NSC19 cells. (**A**–**B**) Ex-4 is neurotrophic in NSC19 cells and pretreatment with Ex-4 protects cells from H_2_O_2_-induced cell death. Cells were pretreated with 100 nM Ex-4 for 4 h, and then exposed to 1.5 mM H_2_O_2_. Cell viability was assessed by MTS (**A**) after 24 h or LDH (**B**) after 48 h (C: vehicle-treated control; Ex-4: exendin-4 with and without H_2_O_2_, n≥6, * p<0.05). (**C**) Ex-4 pretreatment inhibits apoptosis in NSC19 cells. Exposure to 100 nM staurosporine significantly induced caspase-3 activity in NSC19 cells, pretreatment with 100 nM Ex-4 for 24 h reduced elevated caspase-3 activity in these cells (C: vehicle-treated control; Ex-4: exendin-4 with and without staurosporine, n = 3, * p<0.05). **(D**) Ex-4 elevates choline acetyltransferase (ChAT) activity in NSC 19 cells. NSC19 cells plated on 24-well plates were treated with 100 nM or 1 µM of Ex-4 as well as 10 µM forskolin for 72 h. ChAT activity was measured in cell lysates and normalized by protein content (n = 3, * p<0.05).

To elucidate the effect of GLP-1R stimulation on cell viability and neuroprotection against oxidative stress in NSC19 cells, cells were pretreated with 100 nM Ex-4 for 4 h, and H_2_O_2_ was then added to a final concentration of 1.5 mM (established from a dose-response curve, not shown) to induce oxidative stress and cell death. Cell viability was thereafter assessed by MTS and LDH assays at 24 h or 48 h after H_2_O_2_ treatment, respectively. As evident in Figure 2A, 100 nM Ex-4-treatment alone significantly increased cell viability (to 120% of controls as measured in MTS assay at 24 h). In accord, cell membrane permeability integrity, as assessed by LDH release, was significantly improved by Ex-4 treatment alone at 48 h, as compared to controls (Figure 2B). These data signify that GLP-1R pathway activation by Ex-4 has neurotrophic actions in NSC19 cells. Treatment of NSC19 cells with 1.5 mM H_2_O_2_ caused a significant decline in cell viability (a 20% decrease in MTS) and a more than 3-fold loss in membrane permeability integrity (assessed by LDH assay), which were both significantly ameliorated by Ex-4 pretreatment (Figure 2A and 2B), resulting in a restoration in MTS to a level similar to controls and a significant amelioration of the H_2_O_2_-induced LDH elevation. These data suggest that Ex-4-induced stimulation of the GLP-1R pathway provides protection against H_2_O_2_–induced oxidative stress in NSC19 cells. This was assessed further with staurosporine, which induces apoptosis by activating caspase-3, to define whether Ex-4 could similarly ameliorate this action. NSC19 cells were pretreated with 100 nM Ex-4 for 24 h before staurosporine was added at a final concentration of 100 nM. Six hours after initiation of staurosporine treatment, caspase-3 activity was quantified in cell lysates. Caspase-3 activity increased 2.5-fold in staurosporine-treated cells, versus controls, and this increase was significantly attenuated by Ex-4 pretreatment (Figure 2C). These data suggest that Ex-4 has an anti-apoptotic action in NSC19 cells, involving inhibition of caspase-3.

As NSC19 cells express ChAT activity [26], we thereafter assessed whether the GLP-1R pathway could modulate ChAT expression. NSC19 cells were exposed in 24-well plates to 0, 100 nM or 1 µM Ex-4 or 10 µM of forskolin for 48 h or 72 h. ChAT activity was then measured in cell lysates using a radioisotope-based protocol for cultured cells [27]. Ex-4 treatment significantly elevated ChAT activities at both 48 h (not shown) and 72 h (Figure 2D). Forskolin, a positive control that activates adenylyl cyclase and thereby increases intracellular levels of cyclic AMP, also elevated ChAT activity at both times. These data suggest that activation of the GLP-1R pathway may functionally improve cholinergic neurons by promoting acetylcholine production through elevated ChAT activity, which may provide beneficial effects for neurodegenerative diseases such as ALS that involve cholinergic neuron decline.

### Protective actions of Ex-4 in NSC19 cells expressing mutant SOD1

**Figure 3:**
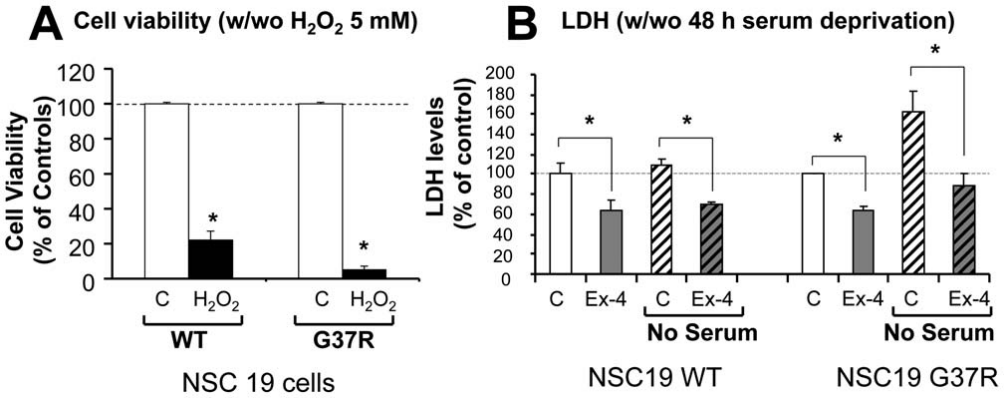
Ex-4 is neuroprotective in NSC19 cells expressing either WT SOD1 or G37R SOD1. (**A**) G37R SOD1 NSC19 cells are more vulnerable to oxidative stress (H_2_O_2_ (5 mM) for 24 h) than are WT SOD1 NSC19 cells. Cell viability assessed by MTS assay shows 22% cell survival in WT SOD1 cells *v.s.* 5% survival in G37R SOD1 cells (n = 6, * p<0.05). (**B**) Ex-4 reduced cell membrane permeability and protected cells from serum deprivation in both WT SOD1- and G37R SOD1-expressing NSC19 cells. Cells were cultured in no-serum media for 48 h in the presence and absence of 100 nM Ex-4. Cell viability was assessed by LDH assay. LDH levels were significantly reduced by Ex-4 treatment in both cells, under conditions of either Ex-4 alone or Ex-4 plus no-serum stress (C: vehicle-treated control; Ex-4: exendin-4; no serum: serum deprivation, n = 5, * p<0.05).

**Figure 4:**
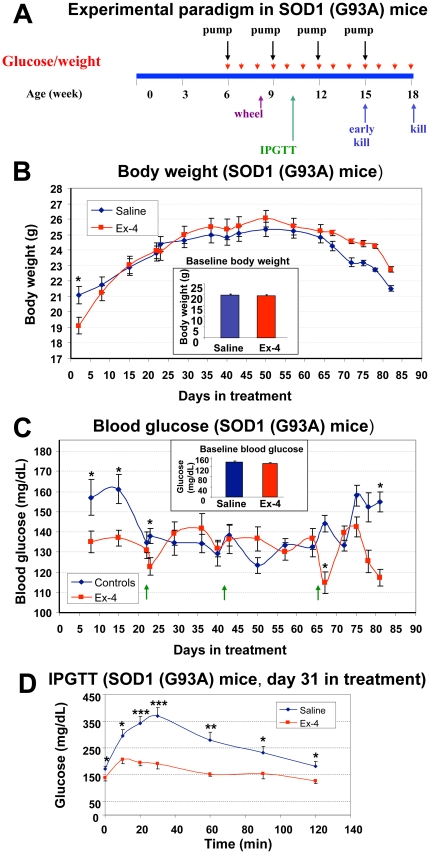
Design of animal study and gluco-regulatory effects of Ex-4 in SOD1 (G93A) mice. (**A**) Scheme of animal study: SOD1 (G93A) mice were assigned to two groups (n = 15 mice/group), starting at the age of 6 weeks, saline or Ex-4 was delivered via subcutaneously implanted osmotic pumps in the control group and Ex-4 treatment group, respectively, throughout the study (treatment duration 12 weeks, hence mice were 18-weeks old at endstage) and pumps were changed every 3 weeks. (**B**) Baseline and weekly body weight of control (saline) and Ex-4-treated SOD1 (G93A) mice were largely similar throughout the study. Both mean baseline and weekly body weights were not different between the two groups of SOD1 (G93A) mice, except at the first measurement on day 2 post-treatment, when the body weight of Ex-4 treated mice transiently dropped compared to controls. The mean body weight of the Ex-4 group thereafter quickly caught up and remained comparable to controls throughout the study (n = 10–15, * p<0.05). (**C**) Baseline and weekly blood glucose levels of saline or Ex-4-treated SOD1 (G93A) mice. Morning fed blood glucose levels were measured weekly from tail tips. In general, blood glucose levels in Ex-4 treated mice were regulated between 120–140 mg/dL, whereas those for controls varied between 125–160 mg/dL. Mean blood glucose levels were largely comparable between the two groups, except during the first 2 weeks and last week of the study, when levels were significantly higher in the control group (n = 10–15, * p<0.05). (**D**) Intraperitoneal glucose tolerance test (IPGTT) at day 31 of treatment in SOD1 (G93A) mice. Assessment of mean blood glucose levels during a 2-hour IPGTT demonstrated significantly lower levels in Ex-4-treated mice at all time points (0, 10, 20, 30, 60, 90, 120 min) measured during IPGTT (n = 7, * p<0.05).

Having established stable NSC19 cell lines that expressed either wild-type (WT) or mutant (G37R) SOD1, we determined that 5 mM H_2_O_2_ treatment for 24 h (established from a dose-response curve, not shown) induced a more significant death of NSC19 cells expressing G37R SOD1 than WT SOD1. As shown in Figure 3A, survival was 5% and 22% in the former and latter, respectively. Likewise, a 48 h serum deprivation induced a significant loss of cell membrane permeability integrity in G37R SOD1 NSC19 cells but not WT SOD1 NSC19 cells, as indicated by elevated LDH levels in the culture media (Figure 4B). These results signify that mutant SOD1 NSC19 cells are more vulnerable to stress, such as induced by oxidative stress and trophic factor withdrawal. Furthermore, Ex-4 not only lowered basal LDH levels in both WT and G37R SOD1-expressing cell lines, as previously seen in control NSC19 cells, but also abolished the serum deprivation-induced elevation of LDH levels in mutant SOD1 NSC19 cells (Figure 3B). Together, these cell culture studies suggest that GLP-1R activation may provide protective effects and ameliorate motor neuron damage in mutant SOD1 animal models of ALS.

### SOD1 (G93A) mouse studies

To define whether the neuroprotective actions of Ex-4 evident in NSC19 cells translate to *in vivo*, we studied the SOD1 (G93A) mouse, a well characterized and widely used animal model of SOD1 mutation-mediated familial ALS [4], [5]. The relationship between mutant SOD1 mouse models, including their advantages/disadvantages, and human ALS has been reviewed [28], . Treatment initiation of SOD1 (G93A) mice began at the age of 6 weeks with either vehicle or Ex-4 via subcutaneous (s.c.) osmotic pump to provide steady-state delivery. Pumps were replaced every three weeks to extend treatment over 12 weeks (Figure 4A) until the occurrence of end stage disease of animals at approximately 18 weeks of age. Consequent to the frailty of the animals, all were euthanized at 18 weeks age. Preliminary studies indicated that Ex-4 remained largely intact over the 3 weeks pump implantation period. In addition, a parallel group of age-matched control WT (B6SJL-Tg(SOD1)2Gur/J) non-ALS mice were studied, bearing the same genetic background to SOD1 (G93A) mice.

### Glucoregulatory effect of Ex-4 in SOD1 mice

To characterize Ex-4 actions on body weight and glucose regulation, blood glucose levels and body weight were measured weekly. Additionally, an intraperitoneal glucose tolerance test (IPGTT) was performed on day 31 of treatment on fasted animals. As illustrated in Figure 4B and inset, baseline values of body weight and blood glucose levels, assessed before initiation of treatment, were not different between vehicle (saline) and Ex-4 SOD1 (G93A) groups. However, mice receiving Ex-4 underwent an initial transient drop in body weight when assessed 2 days after treatment initiation, in line with the known transient action of Ex-4 on food consumption in rodents [30]. This difference was no longer evident one week into treatment and, thereafter, no significant weight differences were evident between the two groups to end stage disease. Morning non-fasting blood glucose levels were initially similar (133 and 138 mg/dL) in both groups of ALS mice, but became transiently elevated in vehicle-treated animals (155–163 mg/dL), likely consequent to stress hyperglycemia associated with osmotic pump implantation. By contrast and as expected, blood glucose levels were unaffected in Ex-4 treated ALS mice, remaining at a baseline of 135 mg/dL, throughout most of the study (Figure 4C). However, during advanced disease a difference in non-fasting blood glucose levels between the vehicle and Ex-4 treated ALS mice again became evident (Figure 4C). To define whether Ex-4 treatment altered glucose tolerance in SOD1 (G93A) mice, an IPGTT was performed midway between pump changes (day 31) in fasted animals at a time that when Ex-4 would be predicted to be at a steady-state concentration. Figure 4D demonstrates that not only did Ex-4 *vs.* vehicle treated ALS mice have a lower resting blood glucose level (zero time), but Ex-4-induced a significant decrease in glucose excursion after an i.p. glucose challenge, providing significantly lowered blood glucose levels at all times measured throughout the 2 h glucose challenge. By contrast, and not shown, age-matched control WT non-ALS mice were normoglycemic.

### Ex-4 treatment normalized running behavior in SOD1 mice

**Figure 5:**
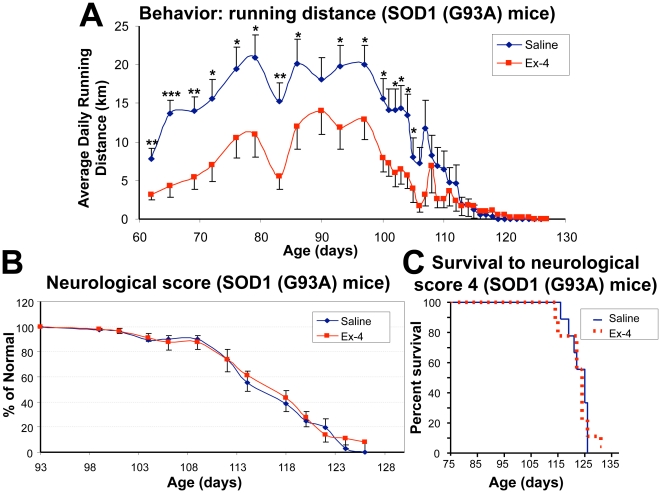
Behavioral effects of exendin-4 action in SOD1 (G93A) mice. (**A**) Comparison of average daily running distances of vehicle-treated and Ex-4-treated SOD1 (G93A) mice. During a presymptomatic stage (approximately 62–95 days of age), Ex-4 treatment significantly reduced average daily running distance of SOD1 (G93A) mice, resulting in 3.2–14.0 km/day (Ex-4 group) *v.s*. 7.8–21.0 km/day (vehicle group) (n = 10–15, * p<0.05, ** p<0.01, *** p<0.001). This Ex-4 induced decline brought the running in line with our prior studies of SOD1 WT non-ALS (B6SJL-Tg(SOD1)2Gur/J) mice [31]. At the age of 95 to 105 days, running activities quickly declined in both SOD1 (G93A) groups in a similar pattern, resulting in disease onset at the same time. (**B**) Neurological scores of SOD1 (G93A) mice. The neurological score between day 93–125 shows no difference between vehicle-treated and Ex-4-treated SOD1 (G93A) mice. (**C**) Kaplan-Meier plots of the survival of vehicle- and Ex-4 treated SOD1 (G93A) mice to reach a neurological score of 4 (paralysis of hind limbs [29]). No difference was evident between the two groups (n = 10 per group, log-rank statistical analysis, p = 0.8).

Our previous study determined that when running wheels are provided in each mouse's cage, presymptomatic SOD1 (G93A) mice are more active runners (15–20 km/day) than age-matched control WT non-ALS (B6SJL-Tg(SOD1)2Gur/J) mice (7–9 km/day) [31]. The SOD1 (G93A) mice then exhibit a sharp decline in daily running distance 10–20 days prior to the onset of clinical disease. In the present study, we compared the running behavior of the two groups of SOD1 (G93A) mice treated with either vehicle or Ex-4 to assess whether GLP-1R activation modifies this behavior. Running distances were recorded twice weekly, from 9-weeks of age to end stage disease. As shown in Figure 5A, Ex-4 treated SOD1 (G93A) mice ran significantly less distance daily (3.2–14.0 km/day) than those treated with vehicle (7.8–21.0 km/day) throughout their presymptomatic disease stage. The vehicle SOD1 (G93A) mice thereby ran 4.1–10 km further daily than their Ex-4 treated littermates, with the latter expressing a running behavior in line with that previously described for control WT mice without ALS. However, the change in running behavior did not impact disease onset, as mean clinical symptom onset dates were 104 days in vehicle SOD1 (G93A) mice and 102 days in Ex-4 treated mice. Using a 5 point neurological score system (see Methodology) to track disease onset and progression, we found no difference between vehicle treated and Ex-4 treated SOD1 (G93A) mice (Figure 5B), and survival time to decline in neurological status to stage 4 was similarly no different (Figure 5C).

### Ex-4 treatment preserved lumbar spinal cord structure, neuron density and specific markers in SOD1 (G93A) mice

**Figure 6:**
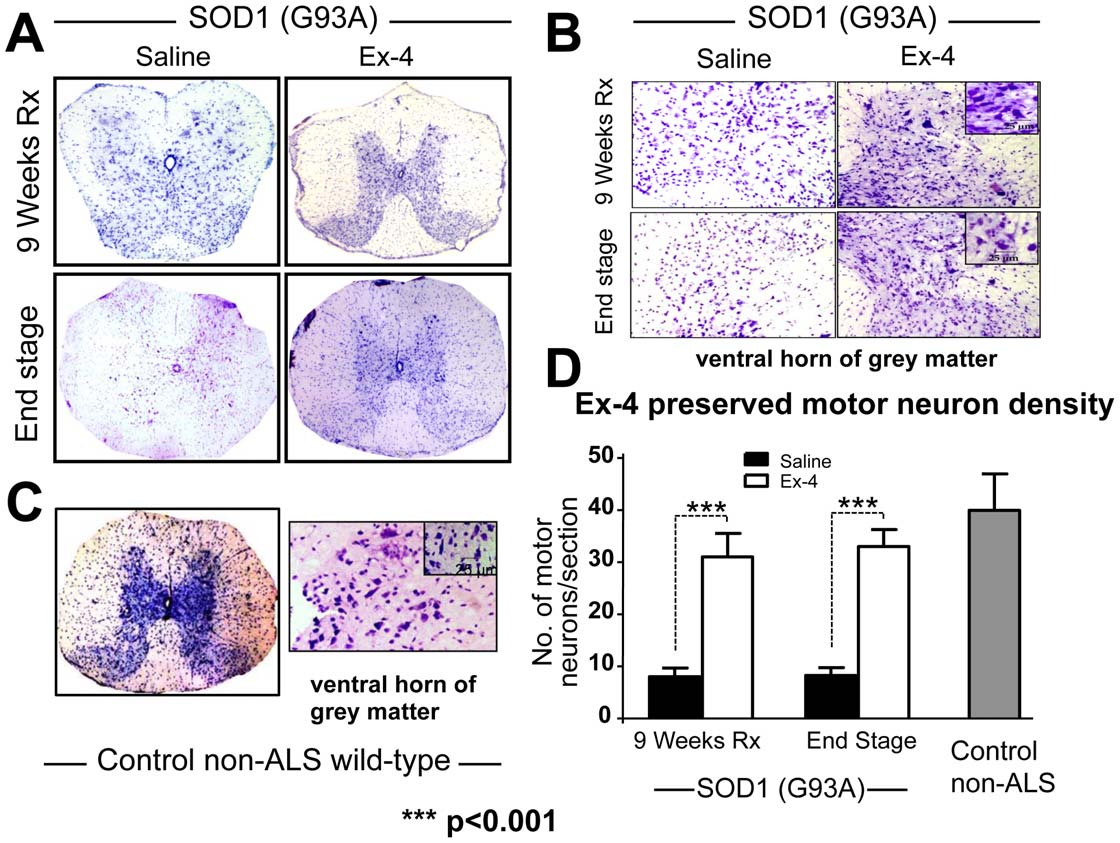
Ex-4 treatment preserved lumbar spinal cord structure and neuron density. (**A**) Cresyl violet staining of lumbar spinal cord section of SOD1 (G93A) mice at both early (9 weeks of treatment at a pre-symptomatic stage) and end stages of disease. (**B**) Close-up of ventral horn of grey matter shows staining of motor neurons with large soma, scale bars on right inset is 25 µm. By comparison, (**C**) demonstrates similar lumbar spinal cord sections derived from control non-ALS WT mice, in which the grey matter tracts are clearly evident. (**D**) Quantification of motor neuron numbers in the ventral horn of grey matter. An approximately 3-fold greater neuron number was present in SOD1 (G93A) Ex-4-treated mice spinal cord sections than in vehicle-treated mice at both pre-symptomatic and end disease stages (9 and 12 week treatment times). Evaluation of motor neuron number in the lumbar ventral horn grey matter of age-matched control non-ALS WT mice were in the range of those present in SOD1 (G93A) mice treated with Ex-4. Selection of large pyramidal neurons was based on their characteristic nulceolus, angular and spindle-shaped [85]. A measuring bar of 25 µm is for reference only.

SOD1 (G93A) mice were euthanized at both 15-weeks (early symptomatic disease stage, n = 5 per group) and 18 weeks of age (end stage, n = 10 per group), and age-matched control WT non-ALS mice (n = 4) were likewise euthanized for analysis of spinal cord. Representative micrographs of cresyl violet-stained lumbar spinal cord are shown in Figure 6A, highlighting the Nissl substance in the neurons. Readily apparent are structural alterations in spinal cord morphology from SOD1 (G93A) vehicle treated animals, particularly in relation to the boundary between white and grey matter regions (Figure 6B), as compared to control wild-type mice (Figure 6C). In particular, the demarcation of the ventral and dorsal grey matter horns is unclear, and suggestive of motor neuron dysfunction or loss within these areas. In contrast, in Ex-4 treated SOD1 (G93A) mice spinal cord, the morphology of the lumbar structure appeared more typical, with both ventral and dorsal horns clearly visible, suggesting preservation of spinal cord structure and neurons (Figure 6A). To more clearly characterize changes, quantification of presumptive motor neuron cell bodies within the ventral horn was undertaken (Figure 6B) to determine neuron density. Figure 6D illustrates the presence of 3-fold more neurons within the spinal cord section of Ex-4 treated SOD1 (G93A) mice compared to vehicle SOD-1 (G93A) mice at both early and end stage disease. This result suggests that Ex-4 treatment preserved lumbar spinal cord neurons from degeneration in ALS mice, particularly since neuron density was reduced but not substantially different from that in control non-ALS mice.

**Figure 7:**
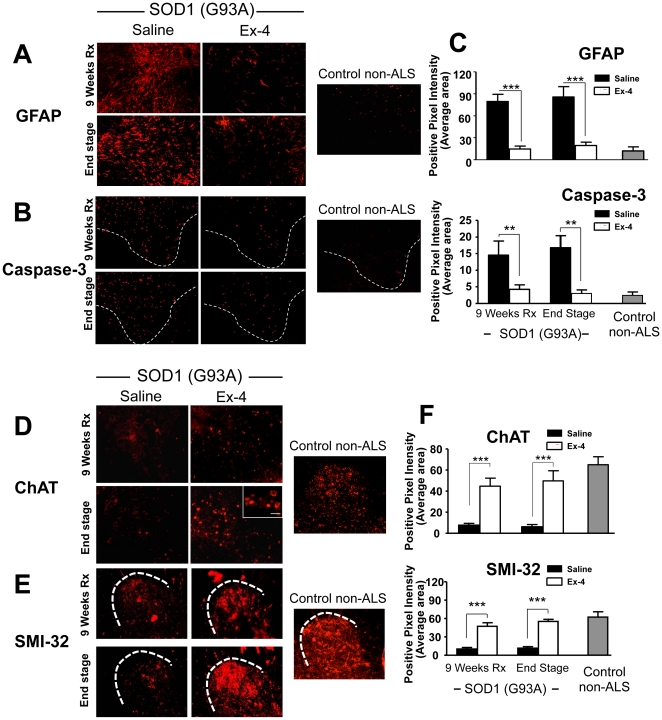
Ex-4 treatment modified cell type specific markers of spinal cord in SOD1 (G93A) mice towards levels present in control non-ALS WT mice. (**A** & **C**) Glial fibrillary acidic protein (GFAP) is a glial cell marker and is usually up regulated under neurodegenerative conditions [86]. Ex-4 treatment significantly reduced GFAP immunostaining in SOD1 (G93A) mice spinal cord. (**B** & **C**) Caspase-3 is an apoptotic marker. There are 3- to 4-fold less numerous activated caspase-3 neurons in Ex-4-treated G93A SOD1 mice lumbar spinal cord sections. (**D** & **F**) Choline acetyl transferase (ChAT) is a cholinergic neuron marker. Immunostaining with specific ChAT antibody shows significantly greater ChAT immunointensity in Ex-4-treated SOD1 (G93A) mice. (**E** & **F**) SMI-32 is a neurofilament marker that interacts with a nonphosphorylated epitope in neurofilament H of most mammalian species. There is greater SMI-32 immunointensity in SOD1 (G93A) mice (n = 5, ** p<0.01, *** p<0.001). For all investigated markers, Ex-4 treatment of SOD1 (G93A) mice substantially ameliorated the dramatic differences evident between vehicle SOD1 (G93A) and control non-ALS WT mice (C & F).

To define whether Ex-4 treatment for 9 and 12 weeks improved function of motor neurons at the cellular level, immunohistochemical staining of spinal cord sections was performed utilizing antibodies against specific markers (Figure 7). Neurodegenerative conditions such as ALS are associated with an up-regulation of glial fribrillary acidic protein (GFAP), a glial cell marker [4], [5]. As shown in Figure 7A, GFAP immunostaining intensity is significantly less in Ex-4 treated SOD1 (G93A) mouse spinal cord than in vehicle treated animals, reaching a difference of approximately 4-fold (Figure 7C). Caspase-3 is an apoptotic marker, and caspase-mediated programmed cell death has been reported as an important mechanism for motor neuron loss in SOD1 (G93A) mice [32]. Utilizing an anti-activated caspase-3 antibody, Figure 7B demonstrates that caspase-3 immunostaining intensity is 3-fold less in Ex-4 treated SOD1 (G93A) mouse spinal cord at early stage (9 weeks Ex-4 treatment) and 4-fold less at end stage disease, as compared to vehicle treatment. These caspase-3 levels in Ex-4 treated SOD1 (G93A) mice approached levels present in control non-ALS WT mice, suggesting that Ex-4 inhibited caspase-3-mediated apoptosis in SOD1 (G93A) mice, and providing a potential mechanism to account for the described motor neuron protection. Choline acetyl transferase (ChAT) is a marker of cholinergic neurons, the primary component of motor neurons. As illustrated in Figure 7D, a substantial decline in ChAT immunointensity was evident within the spinal cord of SOD1 (G93A) vehicle treated mice that was not found following Ex-4 treatment. Five-fold higher levels were present in Ex-4 versus vehicle treated SOD1 (G93A) mice, approaching those found in control non-ALS WT mice (Figure 7F), and supporting an Ex-4 mediated preservation of cholinergic neurons in the spinal cord of treated mice. Finally, SMI-32 is a neurofilament marker to define neuronal cells, interacting with a nonphosphorylated epitope in neurofilament H of most mammalian species. As found for ChAT, a clear decline in SMI-32 immunostaining was evident in the spinal cord of SOD1 (G93A) vehicle, but not Ex-4 treated mice. Levels in the latter approached those present in control non-ALS WT mice and, likewise, suggest a preservation of spinal cord neurons in SOD1 (G93A) mice following Ex-4 treatment (Figure 7E & F).

## Discussion

This study demonstrates that the GLP-1R agonist Ex-4, a well-tolerated drug approved for the treatment for T2DM [7], [8], has a range of beneficial neuroprotective as well as neurotrophic properties not previously described in SOD1 cellular models. Importantly, some of these actions effectively translated from normal and SOD1 (G37R) mutation neuronal cultures into a well-characterized SOD1 (G93A) mouse model of ALS to provide improved glucose tolerance, a preservation of lumbar spinal cord structure and neuron density, an amelioration of the loss of specific spinal cord markers, a concomitant reduction in apoptotic markers in spinal cord, and a change in wheel running behavior towards that of the wild-type. Our data hence suggest that stimulation of the GLP-1R pathway may provide beneficial properties for motor neurons, and therefore warrants further investigation to define its therapeutic potential for the treatment of ALS.

GLP-1R expression has been described throughout the brain, including the hypothalamus, cortex, hippocampus, striatum, substantia nigra and brain stem, as well as subventricular zone [9]–[11]. Under normal physiological conditions, its expression is primarily confined to large output neurons, epitomized by pyramidal and dentate granule neurons as well as Purkinje cells, where, in particular, it localizes to dendrites and on or near synapses [11]. Recent studies have demonstrated that GLP-1R agonists enhance synaptic plasticity [33], [34], strengthening long-term potentiation [33] and improving cognitive performance [35], in accord with GLP-1R over-expression mice exhibiting enhanced learning [36] and knockouts with impaired memory formation [37]. The expression of GLP-1R in spinal cord has been previously shown by in situ hybridization analysis [38], in which scattered GLP-1R mRNA containing cells, that included motor neurons, were apparent within the gray matter. Herein, we directly demonstrate by RT-PCR that GLP-1R mRNA is present within mouse spinal cord as well as in the motor neuron/neuroblastoma cell line NSC 19, implicating a potential function of GLP-1 in motor neurons, and providing support for GLP-1R as a drug target.

In accord with our prior studies in cellular models of AD, stroke and PD in which Ex-4 proved to be neuroprotective against, Aβ-, hypoxia- and 6-hydroxydopamine-induced neuronal toxicity, respectively [15], [16], Ex-4 proved to be protective for NSC 19 cells challenged with oxidative stress at a concentration within a clinically achievable realm [39]. Ex-4, additionally, ameliorated a classical staurosporine-induced apoptotic insult that is known to involve caspase-3 activation, and protected not only NSC19 WT SOD1 but also mutant SOD1 (G37R) expressing cells, which were more vulnerable to oxidative stress, from cellular dysfunction consequent to trophic factor withdrawal. Likewise, and in accord with the Ex-4 induced elevated expression of the dopaminergic marker, tyrosine hydroxylase, in unchallenged primary dopaminergic neurons [15], GLP-1R activation proved to be neurotrophic in unchallenged NSC19 cells, elevating expression of the cholinergic marker, ChAT, and increasing cell viability. The GLP-1 signaling pathway is initiated by Ex-4 and GLP-1 binding to its receptor, a 7-transmembrane protein that belongs to the class B1 G-protein-coupled receptor family [40]. The GLP-1R is associated with adenylyl cyclase that, upon activation, increases intracellular cAMP levels. Downstream of cAMP, many GLP-1 actions have been associated with activation of PKA, PI3K and MAPK [7]. Our prior studies demonstrated that PKA and PI3K mediated pathways are particularly important in the neurotrophic and neuroprotective actions of Ex-4 and GLP-1 in neuronal cultures [15], [16], with MAPK playing a supportive role [14]. GLP-1 actions mediated through MAPK-independent signaling and growth factor–dependent Ser/Thr kinase AktPKB have additionally been described [41]. Interestingly, a recent study in a similar motor neuron cell line, NSC34, describes GLP-1 protection of motor neurons against glucosamine via an Epac-mediated pathway [42]. Thus, similar to pancreatic cells [43], multiple potentially parallel pathways are involved in neuronal trophic/protective actions of GLP-1R activation and it will be interesting to see in future experiments which downstream pathway(s) are most relevant in motor neurons.

A recent study using SOD1 (G93A) mice demonstrated that, compared to WT mice, mutant mice run significantly greater daily distances on a running wheel [31], in line with a documented increased excitability of motor neuron axons in ALS patients [44]. Although disease onset and progression was not correlated with the cumulative running distance of SOD1 (G93A) mice, a clear decline in running distance was readily observed at age 15 weeks that preceded the classical ALS symptomatic stage by 7 to 10 days. In the present study, this likewise proved to be the case, thereby confirming our previous finding. Interestingly, we determined that Ex-4 treatment significantly lowered daily running activity of SOD1 (G93A) mice compared with those administered vehicle, to provide a running behavior in line with WT mice. Albeit the mechanism(s) underlying the increased running phenomenon in SOD (G93A) mice remain unknown [31], the change in behavior provided a valuable pre-symptomatic point to characterize motor neurons at the level of the lumbar spinal cord, and additionally assess the in vivo translation of beneficial cellular actions of Ex-4.

Both GLP-1 and Ex-4 appear to readily cross the blood-brain barrier and enter brain following their systemic administration [12], [13]. In accord with previous reports of beneficial cellular neuroprotective actions of GLP-1R activation translating to improvements in animal models of stroke [15], [18], PD [15], [19]–[21], AD [17], [23], [45], [46] and Huntington's disease [47], a clear amelioration of neuronal loss was evident by immunohistochemical analyses within the ventral horns of the lumbar spinal cord of Ex-4 treated SOD1 (G93A) mice. Specifically, the substantial approximately 80% loss of motor neurons evident both pre-symptomatically and at end stage disease was reduced to a ∼20% loss following Ex-4 treatment, with similar degrees of preservation assessed by quantifying the intensity of both ChAT and the neuronal cell neurofilament protein, SMI-32.

In both human ALS [48] as well as in SOD1 (G93A) mice, reactive astrocytosis is a hallmark of the disease, particularly during the symptomatic phase when hind limb weakness becomes increasingly evident [49]. Our studies indicate the occurrence of astrocytosis, evident from a dramatic elevation of GFAP in the ventral horn of lumbar spinal cord, at both presymptomatic and end stage disease that was largely ameliorated by Ex-4. Accumulating evidence supports a role for astrocytes in disease propagation and advancement of motor neuron dysfunction and subsequent apoptosis mediated by a combination of cell-autonomous and non–cell-autonomous processes [50]–[52]. Astrocytes are clearly critical partners of motor neurons, providing them both trophic support and, via the action of their glial glutamate transporter (EAAT2), rapidly recovering and thereby lowering synaptic glutamate following neuronal excitation. Under conditions where SOD1 (G93A) motor neurons are more vulnerable to physiological stress [51] and SOD1 (G93A) glial cells are less able to support motor neuron survival [51], [52], a depleted expression of EAAT2, resulting in elevated glutamate and potential of excitotoxicity, together with glial cell activation and concomitant release of proinflammatory cytokines, described in cellular and animal models of ALS [53], clearly has greater likelihood to impair the function of neighboring motor neurons and induce apoptosis.

Likewise, interactions involving microglia-motor neuron signaling are implicit in motor neuron health and survival [54], and GLP-1R expression has been described on microglia [55]. Microglia mediate a balance between neuroprotection and cytotoxicity, and whereas resting (M2) microglia provide trophic support, activated (M1) microglia produce and release nitric oxide, superoxide radicals and peroxynitrite that mediate oxidative damage, together with proinflammatory cytokines that can lead to motor neuron injury and death [54]. Activated M1 microglia are an early pathological hallmark of human ALS [56] and occur at a preclinical disease stage in the spinal cord of ALS (G93A) mice [57]. Numerous studies suggest that activated mutated SOD1 microglia more readily switch from survival-promoting to death-promoting with accompanying enhanced release of neurotoxic factors [58], and that excessive oxidation of wild type SOD1 can result in misfolded protein that may acquire the binding and toxic properties characteristic of mutated SOD1 [59], conceptually linking pathways as potentially common between familial and sporadic ALS [60], [54]. Not only does GLP-1R activation provide neurotrophic support and mitigate oxidative stress-induced cellular damage [Figure 2 & 3], but it has been reported to suppress the activation of microglia and inhibit their production of proinflammatory cytokines [55].

The beneficial actions of Ex-4 in SOD1 (G93A) mice are thus potentially mediated at a number of levels, and certainly at the level of improving glucose regulation (Figure 4D) that, similar to human ALS [61], appeared impaired, particularly during advanced disease [62]. Glucose intolerance and insulin dysregulation are evident across a number of neurodegenerative diseases, may contribute to disease progression and are clearly worth ameliorating [63]. As the GLP-1R has as been reported on skeletal muscle [64] as well as on peripheral neurons [65], where its activation is neuroprotective [22], [66], Ex-4 may provide additional peripheral actions in ALS mice at these levels. Moreover, GLP-1R expression has been reported present on glial cells during injury [67], and administration of Ex-4 in models of PD [20], [21] and stroke [18] has been shown to reduce both glial cell activation and levels of proinflammatory cytokines, thereby diminishing neuroinflammation. In addition to the described neuroprotective actions under conditions of oxidative stress, Ex-4 has likewise been shown to be protective against glutamate toxicity [45], and to reduce caspases-3 activation and lower Bax expression [15], [16] via which SOD1 (G93A) motor neuron death has been reported to occur [51], [53]. GLP-1R expression has been demonstrated within the subventricular zone and systemic Ex-4 administration has been reported to stimulate and effectively support neurogenesis [19], which appears deficient in adult ALS models [53], [68]. Furthermore, GLP-1R activation provides neurotrophic actions and not only elevates expression of cholinergic markers and protects neurons from trophic factor withdrawal, but has additionally been reported to promote vascular endothelial growth factor (VEGF) production [69], a trophic protein whose levels are diminished in the plasma and CSF of ALS patients [70] and whose elevated expression appears to improve the survival of SOD1 (G93A) mice [71], [72].

Although Ex-4 clearly demonstrated neuroprotective/neurotrophic motor neuron actions in cellular and animal models of ALS, our in vivo study did not show evidence of symptom free improvement or altered disease progression following Ex-4 treatment (Figures 5B,C), albeit our study was not specifically designed to assess survival. Nevertheless, Ex-4 substantially impacted a number of measures that may translate to quality of life improvements. We cautiously interpret the lack of impact on disease progression by proposing two scenarios, which are not mutually exclusive. First, it is possible that our observed Ex-4-induced preservation of motor neurons may not have been sufficiently physiologically functional to offset neurological deficits, which occur with a particularly steep progression in SOD1 (G93A) mice. The Ex-4 concentrations utilized in our studies are in the realm of those present in human following routine 10 ug subcutaneous Ex-4 administration that achieves plasma levels of 200 pg/ml (48 nM) [39], but higher human doses have been safely administered and could hence be utilized in future preclinical ALS in vivo studies. Second, although alterations in distal motor axons are amongst the earliest pathological changes in the pathogenesis of ALS, followed by a “dying back” process [73], [54], some studies [74] have questioned the origin of ALS and proposed that molecular events occurring within muscle fibers – including oxidative stress, mitochondrial dysfunction and hypermetabolism – may detrimentally impact neuromuscular junction innervation and, thereby, induce motor neuron failure. In this regard, Rouaux and colleagues [75] reported the neuroprotective actions of sodium valproate in ALS mice, mediated via CREB-binding protein dependent mechanisms, which GLP-1 and agonists also have been reported to influence when inducing pancreatic beta cell survival [76], [77]. Sodium valproate, like Ex-4, did not however appear to extend the mean survival of ALS mice [75], potentially because it did not ameliorate skeletal muscle denervation. Whereas the GLP-1R, although potentially different from that present on beta cells [78], appears present and functional in skeletal muscle [64], its existence has yet to be evaluated at the neuromuscular junction. Nevertheless, these results should be viewed in the light of Riluzole, the only approved drug for ALS, that provides a life extension in SOD1 (G93A) of only 10% [79], [80]. Hence future studies of Ex-4 in ALS may warrant assessment of end plate denervation/neuromuscular integrity, nerve conduction time and possibly sciatic nerve diameter to aid characterize physiological function at this peripheral level. In addition, further analysis of Ex-4 following earlier treatment initiation together with a higher Ex-4 dose in SOD1 (G93A) is merited. As may be studies of Ex-4 in the TDP-43 ALS mouse model [81]–[83], which has a slower progression following the occurrence of neurological deficits to potentially allow the assessment of therapy after disease onset. Such studies may further optimize the impact of Ex-4 to allow a clearer determination of clinical translation potential in a disease for which current treatment is clearly unsatisfactory.

## Materials and Methods

### Materials

Peptides (GLP-1 and Ex-4) were obtained from AnaSpec (Fremont, CA). Hydrogen peroxide, staurosporine, forskolin and other reagents were from Sigma (St. Louis, MO). Cell culture media and other cell culture supplements were from Invitrogen (Carlsbad, CA), with the exception of Fetal clone 3 (FC3) serum that was from HyClone (Logan, UT). M-PER cell lysis buffer was from Thermo Fisher Scientific (Rockford, IL).

### Cell cultures

Original NSC-19 cell line was generated by somatic cell fusion of mouse neuroblastoma N18TG2 cells with motor-enriched spinal cord cultures from embryonic day 12–14 mice [26]. Stable NSC-19 cell lines that express wild-type SOD1, mutated SOD1 (G37R) or vector control are established in our laboratory [84]. These NSC-19 cell lines were maintained at 37°C in a 5% CO2 atmosphere in Dulbecco's modified Eagle's medium supplemented with 10% FC3 serum, 100 U/mL penicillin/streptomycin and 0.2 mg/ml G418. Cells were split, 1∶3 ratio, every 3–5 days.

### RT-PCR

Total RNA was extracted from NSC-19 cells cultured on 100 mm dishes by utilizing GenElute ™ Mammalian Total RNA Miniprep Kit from Sigma. RNA quality and quantity were assessed by spectrophotometer at 260 and 280 nM wavelength. One microgram total RNA was used for RT-PCR to identify the presence of the GLP-1R. SuperScript III First-strand Synthesis Supermix (Invitrogen) was utilized for the reverse transcription step, followed by regular PCR. Primers used for PCR were: GLP-1R, forward: 5′ AGGAACCCTACGCTTCGTCAAG 3′ and reverse: 5′ TTTGGCAGGTGGCTGCATACAC 3′, expected PCR product is 250 bp; GAPDH, forward: 5′ TCCACCACCCTGTTGCTGTAG 3′ and reverse: 5′ GACCACAGTCCATGACATCACT 3′, expected PCR product is 452 bp.

### LDH and MTS assays

Cell viability was assessed by LDH (LDH assay kit, Sigma) and MTS assays (CellTiter 96 Aqueous One Solution Cell Proliferation Assay kit, Promega, Madison, WI), as described previously [16].

### Caspase-3 activity

Cellular levels of caspase-3 activity were quantified using a colorimetric caspase-3 assay kit (Sigma) in a 96 well plate microassay, as per the manufacturer's protocol and described previously [16].

### ChAT activity

ChAT activity was quantified utilizing a technique specifically optimized for cell culture studies [27].

### Animal studies

Thirty male B6SJL-Tg(SOD1*G93A)1Gur/J (stock # 002726) mice and four age-matched wild-type control (B6SJL-Tg(SOD1)2Gur/J) mice were purchased from Jackson Laboratories (Bar Harbor, ME) and were housed under controlled 12 hr light/12 hr dark cycle and temperature conditions, with food and water available ad libitum. Animal studies were undertaken on an approved protocol (290-LNS 2013) of the National Institute on Aging Intramural Research Program, and were carried out in strict accordance with the recommendations in the Guide for the Care and Use of Laboratory Animals of the National Institutes of Health. At the start of the study, mice were individually housed and randomly assigned into two groups: group 1: 15 SOD1 (G93A) vehicle mice received saline; group 2: 15 SOD1 (G93A) treatment mice received Ex-4. Starting at the age of six weeks, either saline or Ex-4 were administered to mice via s.c. implanted ALZET Micro-osmotic pumps (Model 1004, Alzet, Cupertino, CA). Ex-4 was dissolved in saline and delivered at a rate of 3.5 pmoles/kg/min. Pumps were changed every 3 weeks for additional 3 times to maintain a steady-state concentration of Ex-4. This thereby provided a total treatment duration of 12 weeks. In all animals, pumps were placed subcutaneously, posterior to the scapulae. Pump implantation and replacement were performed under anesthesia (isoflurane, Abbott Laboratories, Chicago, IL) utilizing sterile procedures. Body weight and blood glucose levels (determined from tail blood by glucometer) were monitored every week. Five mice in both SOD1 (G93A) groups were euthanized at the time of the final (3^rd^) pump replacement when mice were 15-weeks of age and had undergone a total of 9 weeks of treatment. The remaining mice (n = 10) in each group were maintained to end stage disease (defined by a neurological score of 4) and were then euthanized. The wild-type control mice were not treated with Ex-4.

### Intraperitoneal glucose tolerance test (IPGTT)

An IPGTT was performed on day 31 of treatment (approximately midway between pump replacements). Mice were fasted overnight before the glucose tolerance test. Glucose (1.5 g/kg body weight) was administered by i.p. administration. Blood glucose levels were measured by glucometer from samples obtained from the tail vein immediately prior to glucose injection (0 time) and at 10, 20, 30, 60, 90, and 120 min thereafter.

### Behavioral measurement

Mice were individually caged at the start of the study. Two weeks after the initiation of treatment, at 8 weeks of age, mice were transferred to individual cages containing an exercise wheel (Super Pet, IL) coupled to a bicycle computer (Sigma Sport USA, IL). Maximum speed, average speed and total running distance were recorded twice per week, one week later at 9 weeks of age. The motor function of all mice was also scored twice per week using a 5 point clinical observation of the hind limbs with the following stages: 0, normal gait (100%); 1, single leg limp (75%); 2, single leg paralysis (50%); 3, second leg limp (25%); 4, second leg paralysis (0%) [31]. Mice were euthanized on reaching stage 4. Disease onset was defined when the neurological score = 1, whereas disease progression was the rate (days) that it took a mouse to go from a neurological score of 1 to 4. Statistical analyses were performed using Statview software.

### Cresyl violet staining of spinal cord and motor neuron counts

Mice were anesthetized deeply and perfused transcardially with 20 ml filtered cold PBS followed by 20 ml 4% paraformaldehyde. Spinal cords were removed, placed into 4% paraformaldehyde, and kept at 4°C overnight. Cords were then stored for cryoprotection in 30% sucrose overnight. The lumbar spinal cord was removed from the remainder of the spinal cord and cut into 4-mm-long segments. Segments were embedded in OTC freezing medium and sectioned axially at 7 um at −25°C with a Microm Cryostat II, equipped with the Cryo-Jane System (Instrumedics Inc.) for preservation of tissue structure. Every fifth section was stained with 0.1% (w/v) cresyl violet acetate (Nissl stain) without counterstain. Motor neurons in the ventral horn were quantified by counting large pyramidal neurons that stained with cresyl violet and possessed a prominent nucleolus. A minimum of 11 sections of spinal cord were counted per mouse, and all analyses were performed blindly. A minimum of 5 animals per SOD1 (G93A) group were analyzed, and 4 age-matched control wild-type mice were additionally assessed.

### Immunohistochemistry

Mouse spinal cords were fixed, as described above, for the cresyl violet–stained sections. Tissues were exposed for 60 min to PBS containing 0.1% Triton X-100 (Research Organics Inc, Cleveland, OH) and 10% normal goat serum (Sigma, St. Louis, MO) to block nonspecific antibody binding, followed by incubation overnight with primary antibody for the following sets of primary antibodies: (a) Monoclonal Anti-Glial Fibrillary Acidic Protein (1∶200; Sigma, Saint Louis, MO), (b) anti-activated caspase-3 pAb (1∶250, Promega, Madison WI) and (c) ChAT Rabbit pAb (1∶200, Abcam, Cambridge, MA) overnight at 4°C. To test for nonspecific staining by the secondary antibodies, additional slides were processed in a similar fashion with the primary antibodies excluded. All slides were then rinsed for 1 hr at room temperature in several changes of PBS and incubated in the dark for 1 hr at RT in PBS that contained 5% NGS and the fluorescent secondary antibody, Alexa Fluor 568-conjugated IgG (1∶200). Following incubation with secondary antibody, images were acquired by Nikon Eclipse E600 fluorescence microscope. These images were then processed by SPOT advance software, Diagnostic Instruments, Sterling Heights, MI and Photoshop 7.0 (Adobe Systems, San Jose, CA), with the input levels adjusted to span the range of acquired signal intensities exactly.

### Quantification of immunohistochemistry

Using spatially calibrated images with the automated measurement tools in IP lab software (BD Biosciences Bio-imaging, Rockville, MD) total area of positive pixel intensity was measured and analyzed with paired t-test using GraphPad Prism version 5.00 for Windows, GraphPad Software, San Diego California USA.

### Statistical analyses

Data are provided as mean values ± standard error of means. Paired or, where appropriate, unpaired student's t-test and one-way ANOVA were used for statistical evaluation. A Dunnett's t-test was utilized for comparison of multiple samples, with Bonferonni correction as required. The sample number (*n*) together with significance (*p*) and additional statistical analyses are provided in each Figure legend.

## References

[1] Habib AA, Mitsumoto H (2011). Emerging drugs for amyotrophic lateral sclerosis.. Expert Opin Emerg Drugs.

[2] Cozzolino M, Ferri A, Carrì MT (2008). Amyotrophic lateral sclerosis: from current developments in the laboratory to clinical implications.. Antioxid Redox Signal.

[3] Pasinelli P, Brown RH (2006). Molecular biology of amyotrophic lateral sclerosis: insights from genetics.. Nat Rev Neurosci.

[4] Shibata N (2001). Transgenic mouse model for familial amyotrophic lateral sclerosis with superoxide dismutase-1 mutation.. Neuropathol.

[5] Kato S (2008). Amyotrophic lateral sclerosis models and human neuropathology: similarities and differences.. Acta Neuropathol.

[6] Pawlyk AC, Cassel JA, Reitz AB (2010). Current nervous system related drug targets for the treatment of amyotrophic lateral sclerosis.. Curr Pharm Des.

[7] Lovshin JA, Drucker DJ (2009). Incretin-based therapies for type 2 diabetes mellitus.. Nat Rev Endocrinol.

[8] Gallwitz B (2011). Glucagon-like peptide-1 analogues for type 2 diabetes mellitus: current and emerging agents.. Drugs.

[9] Körner M, Stöckli M, Waser B, Reubi JC (2007). GLP-1 receptor expression in human tumors and human normal tissues: potential for in vivo targeting.. J Nucl Med.

[10] Alvarez E, Martínez MD, Roncero I, Chowen JA, García-Cuartero B (2005). The expression of GLP-1 receptor mRNA and protein allows the effect of GLP-1 on glucose metabolism in the human hypothalamus and brainstem.. J Neurochem.

[11] Hamilton A, Hölscher C (2009). Receptors for the incretin glucagon-like peptide-1 are expressed on neurons in the central nervous system.. Neuroreport.

[12] Kastin AJ, Akerstrom V (2003). Entry of exendin-4 into brain is rapid but may be limited at high doses.. Int J Obes Relat Metab Disord.

[13] Banks WA, During MJ, Niehoff ML (2004). Brain uptake of the glucagon-like peptide-1 antagonist exendin(9–39) after intranasal administration.. J Pharmacol Exp Ther.

[14] Perry T, Lahiri DK, Chen D, Zhou J, Shaw KT (2002). A novel neurotrophic property of glucagon-like peptide 1: a promoter of nerve growth factor-mediated differentiation in PC12 cells.. J Pharmacol Exp Ther.

[15] Li Y, Perry T, Kindy MS, Harvey BK, Tweedie D (2009). GLP-1 receptor stimulation preserves primary cortical and dopaminergic neurons in cellular and rodent models of stroke and Parkinsonism.. Proc Natl Acad Sci U S A.

[16] Li Y, Tweedie D, Mattson MP, Holloway HW, Greig NH (2010). Enhancing the GLP-1 receptor signaling pathway leads to proliferation and neuroprotection in human neuroblastoma cells.. J Neurochem.

[17] Li Y, Duffy KB, Ottinger MA, Ray B, Bailey JA (2010). GLP-1 receptor stimulation reduces amyloid-beta peptide accumulation and cytotoxicity in cellular and animal models of Alzheimer's disease.. J Alzheimers Dis.

[18] Teramoto S, Miyamoto N, Yatomi K, Tanaka Y, Oishi H (2011). Exendin-4, a glucagon-like peptide-1 receptor agonist, provides neuroprotection in mice transient focal cerebral ischemia.. J Cereb Blood Flow Metab.

[19] Bertilsson G, Patrone C, Zachrisson O, Andersson A, Dannaeus K (2008). Peptide hormone exendin-4 stimulates subventricular zone neurogenesis in the adult rodent brain and induces recovery in an animal model of Parkinson's disease.. J Neurosci Res.

[20] Harkavyi A, Abuirmeileh A, Lever R, Kingsbury A, Biggs C (2008). Glucagon-like peptide 1 receptor stimulation reverses key deficits in distinct rodent models of Parkinson's disease.. J Neuroinflammation.

[21] Kim S, Moon M, Park S (2009). Exendin-4 protects dopaminergic neurons by inhibition of microglial activation and matrix metalloproteinase-3 expression in an animal model of Parkinson's disease.. J Endocrinol.

[22] Perry T, Holloway HW, Weerasuriya A, Mouton PR, Duffy K (2007). Evidence of GLP-1-mediated neuroprotection in an animal model of pyridoxine-induced peripheral sensory neuropathy.. Exp Neurol.

[23] McClean PL, Parthsarathy V, Faivre E, Hölscher C (2011). The diabetes drug liraglutide prevents degenerative processes in a mouse model of Alzheimer's disease.. J Neurosci.

[24] Hubbard RW, Will AD, Peterson GW, Sanchez A, Gillan WW (1992). Elevated plasma glucagon in amyotrophic lateral sclerosis.. Neurology.

[25] Pradat PF, Bruneteau G, Gordon PH, Dupuis L, Bonnefont-Rousselot D (2010). Impaired glucose tolerance in patients with amyotrophic lateral sclerosis.. Amyotroph Lateral Scler.

[26] Cashman NR, Durham HD, Blusztajn JK, Oda K, Tabira T (1992). Neuroblastoma x spinal cord (NSC) hybrid cell lines resemble developing motor neurons.. Dev Dyn.

[27] Ray B, Simon JR, Lahiri DK (2009). Determination of high-affinity choline uptake (HACU) and choline acetyltransferase (ChAT) activity in the same population of cultured cells.. Brain Res.

[28] Gurney ME (1997). The use of transgenic mouse models of amyotrophic lateral sclerosis in preclinical drug studies.. J Neurol Sci.

[29] Martin LJ (2007). Transgenic mice with human mutant genes causing Parkinson's disease and amyotrophic lateral sclerosis provide common insight into mechanisms of motor neuron selective vulnerability to degeneration.. Rev Neurosci.

[30] Szayna M, Doyle ME, Betkey JA, Holloway HW, Spencer RG (2000). Exendin-4 decelerates food intake, weight gain, and fat deposition in Zucker rats.. Endocrinology.

[31] Bruestle DA, Cutler RG, Telljohann RS, Mattson MP (2009). Decline in daily running distance presages disease onset in a mouse model of ALS.. Neuromolecular Med.

[32] Li M, Ona VO, Guégan C, Chen M, Jackson-Lewis V (2000). Functional role of caspase-1 and caspase-3 in an ALS transgenic mouse model.. Science.

[33] Hölscher C (2010). The role of GLP-1 in neuronal activity and neurodegeneration.. Vitam Horm.

[34] Holst JJ, Burcelin R, Nathanson E (2011). Neuroprotective properties of GLP-1: theoretical and practical applications.. Curr Med Res Opin.

[35] Isacson R, Nielsen E, Dannaeus K, Bertilsson G, Patrone C (2011). The glucagon-like peptide 1 receptor agonist exendin-4 improves reference memory performance and decreases immobility in the forced swim test.. Eur J Pharmacol.

[36] During MJ, Cao L, Zuzga DS, Francis JS, Fitzsimons HL (2003). Glucagon-like peptide-1 receptor is involved in learning and neuroprotection.. Nat Med.

[37] Abbas T, Faivre E, Hölscher C (2009). Impairment of synaptic plasticity and memory formation in GLP-1 receptor KO mice: Interaction between type 2 diabetes and Alzheimer's disease.. Behav Brain Res.

[38] Merchenthaler I, Lane M, Shughrue P (1999). Distribution of pre-pro-glucagon and glucagon-like peptide-1 receptor messenger RNAs in the rat central nervous system.. J Comp Neurol.

[39] Calara F, Taylor K, Han J, Zabala E, Carr EM (2005). A randomized, open-label, crossover study examining the effect of injection site on bioavailability of exenatide (synthetic Exendin-4).. Clin Ther.

[40] Fortin JP, Schroeder JC, Zhu Y, Beinborn M, Kopin AS (2010). Pharmacological characterization of human incretin receptor missense variants.. J Pharmacol Exp Ther.

[41] Perry T, Greig NH (2005). Enhancing central nervous system endogenous GLP-1 receptor pathways for intervention in Alzheimer's disease.. Curr Alzheimer Res.

[42] Lim JG, Lee JJ, Park SH, Park JH, Kim SJ (2010). Glucagon-like peptide-1 protects NSC-34 motor neurons against glucosamine through Epac-mediated glucose uptake enhancement.. Neurosci Lett.

[43] Friedrichsen BN, Neubauer N, Lee YC, Gram VK, Blume N (2006). Stimulation of pancreatic beta-cell replication by incretins involves transcriptional induction of cyclin D1 via multiple signalling pathways.. J Endocrinol.

[44] Vucic S, Kiernan MC (2006). Axonal excitability properties in amyotrophic lateral sclerosis.. Clin Neurophysiol.

[45] Perry T, Haughey NJ, Mattson MP, Egan JM, Greig NH (2002). Protection and reversal of excitotoxic neuronal damage by glucagon-like peptide-1 and exendin-4.. J Pharmacol Exp Ther.

[46] Perry T, Lahiri DK, Sambamurti K, Chen D, Mattson MP (2003). Glucagon-like peptide-1 decreases endogenous amyloid-beta peptide (Abeta) levels and protects hippocampal neurons from death induced by Abeta and iron.. J Neurosci Res.

[47] Martin B, Golden E, Carlson OD, Pistell P, Zhou J (2009). Exendin-4 improves glycemic control, ameliorates brain and pancreatic pathologies, and extends survival in a mouse model of Huntington's disease.. Diabetes.

[48] Schiffer D, Fiano V (2004). Astrogliosis in ALS: possible interpretations according to pathogenetic hypotheses.. Amyotroph Lateral Scler Other Motor Neuron Disord.

[49] Hall ED, Oostveen JA, Gurney ME (1998). Relationship of microglial and astrocytic activation to disease onset and progression in a transgenic model of familial ALS.. Glia.

[50] Boillée S, Vande Velde C, Cleveland DW (2006). ALS: a disease of motor neurons and their nonneuronal neighbors.. Neuron.

[51] Di Giorgio FP, Carrasco MA, Siao MC, Maniatis T, Eggan K (2007). Non-cell autonomous effect of glia on motor neurons in an embryonic stem cell-based ALS model.. Nat Neurosci.

[52] Nagai M, Re DB, Nagata T, Chalazonitis A, Jessell TM (2007). Astrocytes expressing ALS-linked mutated SOD1 release factors selectively toxic to motor neurons.. Nat Neurosci.

[53] Barbeito AG, Mesci P, Boillée S (2010). Motor neuron-immune interactions: the vicious circle of ALS.. J Neural Transm.

[54] Appel SH, Zhao W, Beers DR, Henkel JS (2011). The microglial-motoneuron dialogue in ALS.. Acta Myol.

[55] Iwai T, Ito S, Tanimitsu K, Udagawa S, Oka J (2006). Glucagon-like peptide-1 inhibits LPS-induced IL-1beta production in cultured rat astrocytes.. Neurosci Res.

[56] Henkel JS, Beers DR, Zhao W, Appel SH (2009). Microglia in ALS: the good, the bad, and the resting.. J Neuroimmune Pharmacol.

[57] Dibaj P, Steffens H, Zschüntzsch J, Nadrigny F, Schomburg ED (2011). In Vivo imaging reveals distinct inflammatory activity of CNS microglia versus PNS macrophages in a mouse model for ALS.. PLoS One.

[58] Frankola KA, Greig NH, Luo W, Tweedie D (2011). Targeting TNF-α to elucidate and ameliorate neuroinflammation in neurodegenerative diseases.. CNS Neurol Disord Drug Targets.

[59] Ezzi SA, Urushitani M, Julien J (2007). Wild-type superoxide dismutase acquires binding and toxic properties of ALS-linked mutant forms through oxidation.. J Neurochem.

[60] Bosco DA, Morfini G, Karabacak NM, Song Y, Gros-Louis F (2010). Wild-type and mutant SOD1 share an aberrant conformation and a common pathogenic pathway in ALS.. Nat Neurosci.

[61] Reyes ET, Perurena OH, Festoff BW, Jorgensen R, Moore WV (1984). Insulin resistance in amyotrophic lateral sclerosis.. J Neurol Sci.

[62] Shimizu T, Honda M, Ohashi T, Tsujino M, Nagaoka U (2011). Hyperosmolar hyperglycemic state in advanced amyotrophic lateral sclerosis.. Amyotroph Lateral Scler.

[63] Craft S, Watson GS (2004). Insulin and neurodegenerative disease: shared and specific mechanisms.. Lancet Neurol.

[64] Delgado E, Luque MA, Alcántara A, Trapote MA, Clemente F (1995). Glucagon-like peptide-1 binding to rat skeletal muscle.. Peptides.

[65] Nakagawa A, Satake H, Nakabayashi H, Nishizawa M, Furuya K (2004). Receptor gene expression of glucagon-like peptide-1, but not glucose-dependent insulinotropic polypeptide, in rat nodose ganglion cells.. Auton Neurosci.

[66] Liu WJ, Jin HY, Lee KA, Xie SH, Baek HS (2011). Neuroprotective effect of the glucagon-like peptide-1 receptor agonist, synthetic exendin 4, in streptozotocin-induced diabetic rats.. Br J Pharmacol.

[67] Chowen JA, de Fonseca FR, Alvarez E, Navarro M, García-Segura LM (1999). Increased glucagon-like peptide-1 receptor expression in glia after mechanical lesion of the rat brain.. Neuropeptides.

[68] Lee JC, Jin Y, Jin J, Kang BG, Nam DH (2011). Functional neural stem cell isolation from brains of adult mutant SOD1 (SOD1(G93A)) transgenic amyotrophic lateral sclerosis (ALS) mice.. Neurol Res.

[69] Xiao-Yun X, Zhao-Hui M, Ke C, Hong-Hui H, Yan-Hong X (2011). Glucagon-like peptide-1 improves proliferation and differentiation of endothelial progenitor cells via upregulating VEGF generation.. Med Sci Monit.

[70] Lambrechts D, Storkebaum E, Carmeliet P (2004). VEGF: necessary to prevent motoneuron degeneration, sufficient to treat ALS?. Trends Mol Med.

[71] Azzouz M, Ralph GS, Storkebaum E, Walmsley LE, Mitrophanous KA (2004). VEGF delivery with retrogradely transported lentivector prolongs survival in a mouse ALS model.. Nature.

[72] Wang Y, Mao XO, Xie L, Banwait S, Marti HH (2007). Vascular endothelial growth factor overexpression delays neurodegeneration and prolongs survival in amyotrophic lateral sclerosis mice.. J Neurosci.

[73] Fischer LR, Culver DG, Tennant P, Davis AA, Wang M (2004). Amyotrophic lateral sclerosis is a distal axonopathy: evidence in mice and man.. Exp Neurol.

[74] Gonzalez de Aguilar JL, Echaniz-Laguna A, Fergani A, René F, Meininger V (2007). Amyotrophic lateral sclerosis: all roads lead to Rome.. J Neurochem.

[75] Rouaux C, Panteleeva I, René F, Gonzalez de Aguilar JL (2007). Sodium valproate exerts neuroprotective effects in vivo through CREB-binding protein-dependent mechanisms but does not improve survival in an amyotrophic lateral sclerosis mouse model.. J Neurosci.

[76] Kim SJ, Widenmaier SB, Choi WS, Nian C, Ao Z (2011). Pancreatic β-cell prosurvival effects of the incretin hormones involve post-translational modification of Kv2.1 delayed rectifier channels.. Cell Death Differ.

[77] Dalle S, Quoyer J, Varin E, Costes S (2011). Roles and regulation of the transcription factor CREB in pancreatic β-cells.. Curr Mol Pharmacol.

[78] Luque MA, González N, Márquez L, Acitores A, Redondo A (2002). Glucagon-like peptide-1 (GLP-1) and glucose metabolism in human myocytes.. J Endocrinol.

[79] Gurney ME, Cutting FB, Zhai P, Doble A, Taylor CP (1996). Benefit of vitamin E, riluzole, and gabapentin in a transgenic model of familial amyotrophic lateral sclerosis.. Ann Neurol.

[80] Gurney ME, Fleck TJ, Himes CS, Hall ED (1998). Riluzole preserves motor function in a transgenic model of familial amyotrophic lateral sclerosis.. Neurology.

[81] Wegorzewska I, Bell S, Cairns NJ, Miller TM, Baloh RH (2009). TDP-43 mutant transgenic mice develop features of ALS and frontotemporal lobar degeneration.. Proc Natl Acad Sci U S A.

[82] Wegorzewska I, Baloh RH (2011). TDP-43-based animal models of neurodegeneration: new insights into ALS pathology and pathophysiology.. Neurodegener Dis.

[83] Igaz LM, Kwong LK, Lee EB, Chen-Plotkin A, Swanson E (2011). Dysregulation of the ALS-associated gene TDP-43 leads to neuronal death and degeneration in mice.. J Clin Invest.

[84] Pedersen WA, Cashman NR, Mattson MP (1999). The lipid peroxidation product 4-hydroxynonenal impairs glutamate and glucose transport and choline acetyltransferase activity in NSC-19 motor neuron cells.. Exp Neurol.

[85] Pendersen WA, Mattson MP (1999). No benefit of dietary restriction on disease onset or progression in amyotrophic lateral sclerosis Cu/Zn-superoxide dismutase mutant mice.. Brain Res.

[86] Middeldorp J, Hol EM (2011). GFAP in health and disease.. Prog Neurobiol.

